# Healthcare system barriers and facilitators to hypertension management in Ghana

**DOI:** 10.5334/aogh.4246

**Published:** 2024-07-04

**Authors:** Samuel Byiringiro, Thomas Hinneh, Joylline Chepkorir, Tosin Tomiwa, Yvonne Commodore-Mensah, Jill Marsteller, Fred S. Sarfo, Martha A. Saylor, Shadrack Assibey, Cheryl R. Himmelfarb

**Affiliations:** 1 Johns Hopkins University School of Nursing, Baltimore, Maryland, United States; 2 Johns Hopkins Institute for Clinical and Translational Research; 3 Johns Hopkins University, Bloomberg School of Public Health, Baltimore, Maryland, United States; 4 Kwame Nkrumah University of Science & Technology, Department of Medicine, Kumasi, Ashanti Region, Ghana; 5 Komfo Anokye Teaching Hospital, Kumasi, Ashanti Region, Ghana

**Keywords:** hypertension, high blood pressure, Sub-Saharan Africa, health system

## Abstract

*Background:* Hypertension continues to pose a significant burden on the health systems in Sub-Saharan Africa (SSA). Multiple challenges at the health systems level could impact patients’ blood pressure outcomes. There is a need to understand the gaps in health systems to improve their readiness to manage the rising burden of hypertension

*Objective:* To explore health system barriers and opportunities for improved management of hypertension in Ghana, West Africa.

*Methods:* We conducted 5 focus group discussions involving 9 health facility leaders and 24 clinicians involved in hypertension treatment at 15 primary-level health facilities in Kumasi, Ghana. We held discussions remotely over Zoom and used thematic analysis methods.

*Results:* Four themes emerged from the focus group discussions: (1) financial and geographic inaccessibility of hypertension services; (2) facilities’ struggle to maintain the supply of antihypertensive medications and providers’ perceptions of suboptimal quality of insured medications; (3) shortage of healthcare providers, especially physicians; and (4) patients’ negative self-management practices. Facilitators identified included presence of wellness and hypertension clinics for screening and management of hypertension at some health facilities, nurses’ request for additional roles in hypertension management, and the rising positive practice of patient home blood pressure monitoring.

*Conclusion:* Our findings highlight critical barriers to hypertension service delivery and providers’ abilities to provide quality services. Health facilities should build on ongoing innovations in hypertension screening, task-shifting strategies, and patient self-management to improve hypertension control. In Ghana and other countries, policies to equip healthcare systems with the resources needed for hypertension management could lead to a high improvement in hypertension outcomes among patients.

## Introduction

Hypertension continues to pose a significant burden on the health systems in Sub-Saharan Africa (SSA). Approximately 25% of the population in SSA has hypertension, and only 5% has achieved controlled blood pressure (BP) [1, 2]. When not adequately managed, hypertension can increase the risk of stroke and cardiovascular diseases [3]. However, more than 80% of countries in SSA are unable to offer a minimum of three physician visits per year for optimal BP control, as recommended by the latest clinical guidelines [4, 5]. Well-equipped healthcare systems coupled with population-level interventions to raise awareness about hypertension and improve healthy lifestyles may reduce the burden of hypertension.

In Ghana, 27% of adults have hypertension and only 6% have achieved controlled BP [6]. Similarly to many other SSA countries, more than 60% of people with hypertension in Ghana are yet to be screened and roughly 80% are to be initiated on treatment [6]. In 2014, the Pan-African Society of Cardiology (PASCAR) identified ten steps to achieve 25% hypertension control by 2025 [7]. The 10 steps addressed significant known barriers to hypertension management, including making hypertension management a national priority and funding allocation to facility-level activities such as availability of essential medications, treatment guidelines, and provider training [7]. These steps addressed population-based hypertension management interventions to minimize salt consumption. To ensure objectivity, the 10 steps recommended instituting a monitoring and evaluation system for all the above activities [7]. Nonetheless, a recent study in Ghana reported poor adherence to PASCAR recommendations and noted that there remains a long way to go before achieving the goal of 25% hypertension control [8].

According to the World Health Organization (WHO), to achieve improved health outcomes, health systems must provide social and financial risk protection and be responsive and efficient [9]. In its health systems framework, WHO recommends that nations invest in six key areas: service delivery, the health workforce, health information systems, access to essential medicines, financing, and leadership and governance [9]. As recommended in PASCAR’s 10 steps, improving hypertension management in each of the WHO health system framework areas requires exploring context-specific measures [7]. However, limited tools are available to accurately map health system interventions for managing different health conditions, such as hypertension. The available measurement instruments lack comprehensiveness in integrating all elements of health systems, and different elements require different evaluation methods.

Previous literature has focused on patient-level factors of hypertension outcomes and relatively little on potentially effective health system interventions. While we have ample understanding of the associations between worsening hypertension outcomes and some patient biosocial factors such as increasing age, male sex, low income, and lack of education, and behavioral factors such as smoking, high salt consumption, and sedentary lifestyles [10, 11], the understanding of the role of different health system factors on hypertension outcomes is limited. To address this gap, this study aimed to assess health facility leaders’ and clinicians’ perspectives on the health system barriers and facilitators of hypertension management in Ghana. The findings of this study are essential to inform policies and clinical interventions to address the burden of hypertension.

## Methods

### Setting and population

This study was conducted in Kumasi, the capital city of the Ashanti Region of Ghana. According to the 2021 population census, Kumasi had a population of approximately 3,490,000 people [12] and was served by 263 health facilities. Of all the health facilities, 224 were privately owned, 18 were quasi-governmental, 16 were government-owned, and 5 were owned by the Christian Health Association of Ghana (CHAG) [13]. Together with a local cardiologist and research expert (FSS), we purposively identified and recruited 25 health facilities that offered hypertension screening, diagnosis, treatment, or patient follow-up. Health facilities were at different levels of the health system and had varied ownership. After recruitment, 15 leaders of health facilities signed the Memorandum of Understanding, accepting to join the study. The study population comprised of health facility leaders and clinicians.

### Design

To understand the context of hypertension care, we used a descriptive qualitative design. We held 5 focus group discussions with 7 to 10 individuals each. These included three focus group discussions with the clinicians and two with health facility leaders.

### Study participants

At least 2 clinicians and 1 healthcare facility leader were invited from each of the 15 facilities. Clinicians were eligible to participate in focus group discussions if they were physicians, physician assistants, nurses, or other clinical staff members involved in the management of patients with hypertension.

### Recruitment

Prior to the focus group discussions, we administered quantitative surveys as part of the Addressing Hypertension Care in Africa Project’s (ADHINCRA’s) assessment of multilevel health system determinants of hypertension care and outcomes at 15 health facilities in Kumasi [14, 15]. In a self-administered survey distributed to 67 healthcare providers, we assessed their knowledge of and attitudes toward hypertension treatment guidelines [14]. In this survey, we asked them about their willingness to participate in a focus group discussion regarding their perceptions of barriers to the healthcare system and facilitators of hypertension care. We followed up with participants who expressed interest. We recruited healthcare facility leaders on site.

### Development of focus group discussion guide

The WHO health system framework [9] informed the development of the focus group discussion interview guide (Supplementary File 1: Focus Group Discussion Guide). In reference to the framework, the six building blocks of health systems constituted the key topics of the discussion: service delivery, healthcare providers, medicines, tools and equipment, health information, and leadership and governance. Further, the quantitative findings from the ADHINCRA’s assessment of the multilevel healthcare system determinants of hypertension care and outcomes in the same 15 health facilities helped inform the probing of questions in the focus group discussion guide [14].

## Data Collection

The focus group discussions explored barriers and opportunities in the healthcare systems for managing patients with hypertension. The investigator conducted a 15-minute presentation of aggregated findings from quantitative surveys that assessed patient-, provider-, and health facility–level factors of hypertension care and outcomes in the participants’ health facilities [14]. After the presentation, two trained research assistants from Ghana (TH, SA) followed a semi-structured guide to moderate the discussions. At the end of the focus group discussions, a cardiologist (SS) helped answer technical hypertension management questions clinicians may ask.

While all participants understood English, we held focus group discussions in both English and Twi (a local language spoken in Ghana) to ensure that participants express themselves comfortably. We conducted discussions over Zoom, activating an audio recording option to facilitate data management and analysis. Discussions were 1.5 hours on average each.

### Data management and analysis

After conducting the focus group discussions, two bilingual speakers of English and Twi performed the data transcription and translation of Twi to English. We used Atlas.ti software version 22.2.3 to organize data for analysis [16]. We utilized deductive coding using preset codes in reference to the WHO health systems framework and inductive coding allowing for additional codes if they emerged during the coding process. To promote credibility in the coding process, at least two individuals coded each transcript independently, and a group of four coders convened to discuss any discrepancies. We solved discrepancies by referring to the definitions of the codes or creating new codes if necessary. We reported the findings in themes and provided representative quotes for each theme.

### Ethics statement

The Institutional Review Boards at Kwame Nkrumah University of Science and Technology School of Medicine and Dentistry (Ref: CHRPE/AP/021/22) and Johns Hopkins Medicine (IRB00218586) reviewed and approved this study. All potential participants received an explanation about the study and discussion topics, had chances to ask questions about their involvement in the study, and gave verbal consent before initiating the focus group discussion. To maintain participants’ confidentiality, we encouraged them not to use their real names on the video conferencing platform or during the discussions and encouraged them to deactivate the video throughout the discussion and mute themselves when not speaking. Furthermore, we removed participants’ names from the transcripts before analysis and reported themes using the generic terms “Physician” or “Registered Nurse” instead of individual participants’ names.

## Results

We held 5 focus group discussions involving 9 healthcare facility leaders and 24 clinicians (Table 1). Most health facility leaders were male (7 of 9) and physicians (7 of 9), whereas most clinicians were female [15 (62.5%)] and nurses [18 (75.0%)].

**Table 1:** Characteristics of participants in the Focus Group Discussion.

PARTICIPANTS CHARACTERISTICS	CLINICIANS (N = 24)	HEALTH FACILITY LEADERS (N = 9)
**Sex**		
Male	9 (37.5%)	7 (77.8%)
Female	15 (62.5%)	2 (22.2%)
**Profession**		
Physicians	5 (20.8%)	7 (77.8%)
Nurses	18 (75.0%)	2 (22.2%)
Dietitian	1 (4.2%)	
**Ownership of participants’ health facility**		
Private	9 (37.5%)	4 (44.5%)
Government	11 (45.8%)	4 (44.4%)
CHAG	4 (16.7%)	1 (11.1%)
**Health facility department**		
Outpatient department	14 (58.3%)	N/A
Hypertension clinic	7 (29.2%)	N/A
Others	3 (12.5%)	N/A
CHAG: Christian Health Association of Ghana		

**Figure 1:**
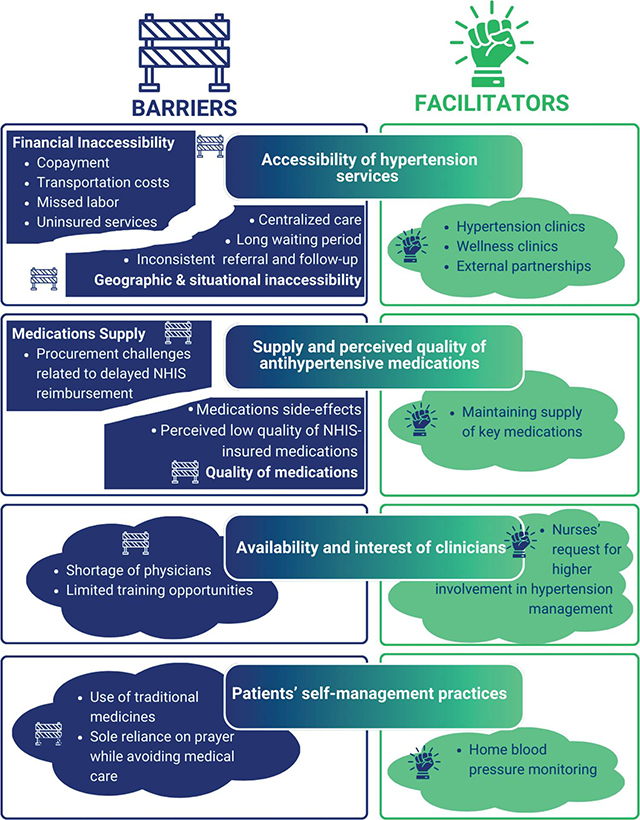
Themes Emerging from the Focus Group Discussion About Barriers and Facilitators to Hypertension Management in Ghana (NHIS: National Health Insurance Scheme).

Four themes classified in barriers and facilitators emerged from the focus group discussions (Figure 1): (1) the challenges surrounding financial, geographic, and situational accessibility of hypertension services; (2) facilities’ struggle to maintain the supply of antihypertensive medications and providers’ perceptions of suboptimal quality of insured medications; (3) the shortage of clinicians and nurses’ request for higher involvement in hypertension management; and (4) patients’ positive and negative self-management practices. Supplementary File 2 provides additional details on the subthemes and selected representative quotes (Supplementary File 2).

### Theme 1: The financial, geographic, and situational accessibility of hypertension services

Participants described the high cost of care as a major factor in low healthcare utilization and loss to follow-up among patients with hypertension. In addition to paying annual health insurance premiums, patients are required to pay additional fees (often called “top-ups”) for insurance-covered services, medical procedures, or products. Clinicians and facility leaders indicated that there are antihypertensive medications that are not covered by the National Health Insurance Scheme (NHIS), and when these are the only available medications at the health facility, patients are required to pay the full out-of-pocket cost. Similarly, clinicians noted that regularly prescribed laboratory examinations and imaging procedures were not covered by NHIS. Despite the high cost of care, there is no financial support system for patients who cannot afford it.

*When you want to run labs for them too, there is no money, so you are not able to. And at times, a complication will develop since the person did not do the labs for us to detect things early.*

*—Registered Nurse*

Geographically, hypertensive services are not readily accessible**.** The participants reported that the screening was mainly held at health facilities and that only one or two days a week were dedicated to hypertension care. Patients who travel long distances often struggle to receive care and sometimes come to the health facility, spend all day, and are still not seen by specialists. Additional geographic accessibility challenges result from inconsistent referrals and patient follow-up systems.

*The thing is, some come from different regions, so sometimes we don’t get to attend to them because of the place they are coming from. That one too is a factor in my facility. Some leave there around 10 pm, by that time too, the cardiologist will say I am tired. I must go. So, they wouldn’t even see the cardiologist.*

*—Registered Nurse*

On a positive note, some health facilities have specialized units that manage patients with hypertension, referred to as “hypertension clinics.” The waiting time for patients who receive care at facilities with hypertension clinics is shorter, and patient educational sessions are more structured than for patients who receive care in the general outpatient department. Moreover, while the screening in the other health facility departments target people seeking healthcare services, some health facilities have special units called “Wellness Clinics,” where every individual coming to the health facility as a patient, patient accompanier, caregiver, or just a passerby can go to have their BP measured free of charge and potentially initiated on treatment if found to have elevated BP. Additionally, health facilities working with Healthy Heart Africa [17], a non-government organization providing support for hypertension management, reported receiving support from community outreach events to screen patients with hypertension.

### Theme 2: Facilities struggle to maintain the stock of antihypertensive medications and providers perceive government insured medications to be of suboptimal quality

The participants’ descriptions of the availability of antihypertensive medications were entangled with the reimbursement by the main payor, NHIS; the main supplier of medications for government health facilities is the regional medical store, whereas private health facilities receive their medications from private suppliers. Clinicians rarely reported running out of stock for antihypertensive medications, but leaders reported a challenging process in maintaining optimal quantities of essential antihypertensive medications and other key supplies in the pharmacy.

*You stock the medicines, and if reimbursements are also much delayed, then you have to find means of getting money to stock more of the medicines to supply the clients.*

*—Physician and leader of a private health facility*

An additional challenge is the perceived suboptimal quality of antihypertensive medication. Clinicians have reported that medications insured by the NHIS are often cheap and associated with numerous adverse effects.

*Most of the insurance drugs, because of the low quality it has, patients, most of them react to it, its adverse effect.*

*—Hypertension Clinic Registered Nurse*

### Theme 3: The shortage of clinicians especially physicians and the nurses’ plea for training and additional roles in hypertension management

The common theme across focus group discussions with clinicians and health facility leaders is that the number of healthcare providers, especially physicians and specialists, is extremely inadequate compared to the number of patients they serve, and that this has a negative impact on the quality of hypertension care services. Clinicians are forced to rush their encounters with patients without taking sufficient time to understand the patient’s history or acquire a clearer picture of the patient’s condition before assigning a diagnosis and treatment or renewing a prescription for antihypertensive medication. This situation is frustrating for both the patients and clinicians.

*Sometimes, because of the number of people you must see in a day, there are some people who just come, and you ask if there are any complaints. Some of our colleagues will just take it as if they [patients] came for a refill of their medication without having a critical look at the BP of the patient.*

*—Physician*

While participants reported using clinical meetings and ward rounds as opportunities for learning and sharing knowledge about hypertension care, regular training opportunities on the latest advances in hypertension management are scarce. Due to the shortage of physicians, the participants recommended training more nurses in hypertension management. After a few training sessions, the nurses felt detached because the learning materials were not tailored to their scope of practice. They were eager to take on additional roles in hypertension management and requested training sessions tailored to their educational needs.

### Theme 4: Positive and negative patients’ self-management practices

The positive patient self-management behaviors discussed were home BP monitoring and religious practice. Some patients had BP devices to monitor their own BP at home, and some private pharmacies where patients picked up medications had devices to check BP. During hypertension management, participants relied on the patients’ home BP measures to understand the effect of the medications and inform them of the course of action in hypertension treatment. As part of the recommendation, clinicians and health facility leaders believed that if all patients had access to BP devices at home, it would be beneficial for their BP outcomes.

The harmful self-management practices included reliance on traditional medicine, hence delaying seeking care and, instead of taking antihypertensive medications and following medical advice on healthy behaviors, some patients continued to take traditional recipes.

*They have some myths that herbal preparation can cure it, so they come, you give them the drugs, they go and dash it somewhere, and they are taking the herbal medication.*

*—Physician and leader of a private health facility*

## Discussion

This study aimed to explore healthcare system barriers and facilitators of hypertension care in Kumasi, Ghana, from the perspective of health facility leaders and clinicians. The focus group discussions unveiled key challenges in hypertension service delivery, including challenges in financial and geographic accessibility of hypertension services and some opportunities, including partnership with Healthy Heart Africa and the use of hypertension clinics and wellness clinics to improve hypertension screening. Furthermore, we identified a dire shortage of healthcare providers, especially physicians; the perception of suboptimal quality of medications; and positive and negative patient self-management practices.

Currently, hypertension care in Ghana is primarily centralized at district-level health facility settings with very limited hypertension education, screening, and treatment in the community. Even wellness clinics, a novel strategy of hypertension screening, are run at health facilities. If Ghana is to screen and diagnose the estimated 60% of adults with hypertension [6], awareness campaigns and screening will need to be decentralized closer to the community, preferably at sites frequented by adults on a regular basis. A study in Tunisia screened 1,536 people and educated them about hypertension in the barbershop and reported a 28.5% prevalence of hypertension [18]. In this study, 57% of people screened were men and only 9% of the people identified to have hypertension had previously been diagnosed, demonstrating the power of community-level screening in engaging people who do not frequent health facilities on a regular basis [18]. Additional places to meet adults for hypertension screening and education could include churches, community gatherings, pubs, and shopping malls. These screening and education activities could be spearheaded by the Community-Based Health Planning and Services (CHPS), the program of Ghana Health Service that provides primary healthcare services [19]. Further, the CHPS could be equipped, supported, and empowered to follow up patients with nonresistant hypertension, making hypertension services geographically accessible.

Health facilities struggle to maintain the quality of hypertension care services because of a shortage of healthcare providers, especially physicians. The shortage of physicians is a critical challenge in the management of chronic and non-chronic noncommunicable disease (NCD) in all countries, especially in developing countries. Previous quantitative and qualitative studies in Ghana have pointed out similar challenges of human resource constraints [20, 21]. Human resource shortages result in long waiting periods for patients, rushed consultations that risk medical errors, and ultimately, poor quality of care and low patient satisfaction. Studies have demonstrated that shifting tasks historically conducted by physicians to vital professionals, such as nurses, is effective in resource-constrained settings [21–24]. In settings where it was tried for hypertension management, task shifting was acceptable and appreciated by patients and healthcare providers [21–24]. However, policymakers in Ghana are yet to take up and reinforce task shifting as a new way of managing patients with hypertension.

While clinicians reported that the supply of antihypertensive medications is not a big issue, facility leaders and previous literature pointed this to be a critical challenge for patients with hypertension. A study conducted in the largest referral hospital in Ghana reported that only 15% of patients with hypertension reported receiving their medications from a hospital pharmacy [25]. Another study conducted at five hypertension and diabetes specialty clinics, including Komfo Anokye Teaching Hospital in Kumasi, reported that 23% of patients with elevated BP experienced problems obtaining antihypertensive medications; the main challenge being the unavailability of the medications at the point of care [26]. In our study, health facility leaders often pointed to the lack of supplies at Regional Medical Stores and the delayed reimbursement by the NHIS as the root cause of the problem with medication supply. If not addressed, inconsistency in the supply of medications will continue to reduce the quality of hypertension care and BP outcomes.

Apart from antihypertensive medications, clinicians and health facility leaders voiced major concerns regarding the overall high cost of hypertension care. The high cost of care creates a barrier to hypertension service accessibility, which negatively affects the BP outcomes. Studies in Ghana have reported similar issues with the high cost of hypertension care essentially driven by the high out-of-pocket spending from “top-ups” on medications, laboratory investigations, and transport, including among patients covered by NHIS [21,27]. While the NHIS helped diminish catastrophic healthcare expenditures, it did not eliminate out-of-pocket spending [28]. According to Ghana literature, receiving medicines fully covered by health insurance is associated with two times higher odds of achieving BP control than making any out-of-pocket payments [25] The changes in the NHIS policy require the intervention of high-level policymakers, and it is time they look to the available evidence and act accordingly.

An additional financing-related issue that resurfaced in the current study was the lack of funding for hypertension management activities. Resource scarcity is a common challenge in many developing countries, and most shortcomings in health systems are often attributed to a lack of funds, even though the strategic utilization of available funds is equally important. In 2022, Ghana released the second edition of the National Policy for NCDs, including hypertension-related conditions, following the first edition in 2012 [29]. According to a study evaluating Ghana’s adherence to PASCAR recommendations (which include many strategies that were included in the 2012 NCDs policy), Ghana is still behind in terms of implementing changes that could ultimately improve BP outcomes for patients [8]. The government of Ghana should increase healthcare spending from the current 3.5% to at least 15% of the gross domestic product (GDP), as it pledged in the 2001 Abuja Declaration [30, 31], to fund projects on its health agenda and ensure strategic allocation and accountability.

The patients’ self-management practices are an important element of hypertension outcomes among patients in Ghana. Previous literature has shown that some patients in African countries, including Ghana, opt for traditional medicine instead of seeking or adhering to medical treatment for hypertension [32]. This practice is concerning because the effects of herbal medicine on hypertension have not been thoroughly explored, and there may be interactions between herbal and modern medicines that could have negative effects. According to the study in 12 African countries, the use of traditional medicine was associated with higher rates of severe hypertension complications from hypertension [32]. The weak health systems are partially responsible for the patients’ use of traditional medicine. When the health systems fail to meet the needs of the population through accessible health services, patients seek alternatives, including the reliance on faith through prayer and traditional healers. Future interventions on hypertension should focus on increasing the population awareness of hypertension and available medical treatment as well as promoting positive self-management practices such as home blood pressure monitoring. Also, the combination of nonmedical practices, such as faith-based approaches, and modern medicine has proven to be effective in hypertension management [33]. Leveraging the trust in faith-based organizations can help build sustainable and culturally acceptable interventions for hypertension management in Ghana. Additionally, future studies should demonstrate the safety and effectiveness of the traditional medicine in hypertension management rather than utterly rejecting or welcoming them without robust scientific evidence to back the decision.

## Conclusion

We explored the health system barriers and facilitators of hypertension care in Ghana. Overall, we found accessibility challenges related to hypertension screening, diagnosis, treatment, and a lack of funding for patient follow-up. We also found that health facility leaders struggled to maintain medication storage, and there was a shortage of physicians. Despite these challenges, there are opportunities to capitalize on, including the wellness clinics, which allow the public to receive free screening for hypertension; nurses’ willingness to learn and take up additional roles in hypertension management; and some patients’ positive self-management attitudes, which, if leveraged, could lead to improved hypertension outcomes. To halt the rising burden of hypertension in Ghana and other countries in SSA, policies should strive to arm health systems with the tools and resources needed for the early detection, treatment, and follow-up of patients with hypertension.

## Additional File

The additional file for this article can be found as follows:

10.5334/aogh.4246.s1 Supplementary File 1. Detrended Oscillation and Clock Parameters.

10.5334/aogh.4246.s2 Supplementary File 2. Table 1. Themes, subthemes, and selected quotes on health system barriers and facilitators of hypertension care in Ghana.

## Figures and Tables

**Table:** 

ADHINCRA	Addressing Hypertension Care in Africa
BP	blood pressure
CHAG	Christian Health Association of Ghana
CHPS	Community-based Health Planning and Services
NHIS	National Health Insurance Scheme
PASCAR	Pan-African Society of Cardiology
SSA	Sub-Saharan Africa
WHO	World Health Organization

## Data Availability

All data is available for sharing with a reasonable request to the corresponding author SB (sbyirin1@jhu.edu).
