# Supplementary material for: Healthcare system barriers and facilitators to hypertension management in Ghana
Source: Ann Glob Health. 2024 Jul 4;90(1):38. doi: 10.5334/aogh.4246 (PMC11229483; doi:10.5334/aogh.4246)
Supplement: Supplementary File 1. — Detrended Oscillation and Clock Parameters. [file agh-90-1-4246-s1.pdf]

Supplementary File 1. FOCUS GROUP DISCUSSION INFORMATION AND GUIDE

Thank you for accepting to join this focus group discussion (FGD). This FGD is part of the ADHINCRA's project of exploring the health system determinants of hypertension care and outcomes in Ghana. As part of this project, we recruited 15 health facilities, 67 healthcare providers, and 225 patients with hypertension in Kumasi. We collected data regarding health facilities' and healthcare providers' readiness to manage hypertension effectively. We assessed providers' knowledge of hypertension treatment guidelines, their confidence in adhering to those guidelines, and the use of continuous quality improvement strategies for better management of hypertension. With this FGD, we will understand better and contextualize quantitative findings.

Procedure

You will get a summary of quantitative findings, then discuss with fellow healthcare providers or health facility leaders your experiences in managing patients with hypertension and your perceived health system barriers to and opportunities for better management of hypertension.

Every participant's contribution to the discussion is valuable given your respective roles in managing hypertension at your health facilities. We encourage every participant to engage in the conversation actively, freely voice their ideas, and respect everyone else's.

The FGD is 1 hour and **will be recorded** to facilitate data management, analysis, and reporting of the findings.

Zoom Functions

This discussion is being held over Zoom as opposed to an in-person interaction. We will demonstrate specific Zoom functions to ensure a seamless experience. We encourage you to sit in a quiet place/office to ensure your ideas are well heard.

Zoom allows you to mute/unmute yourself and activate/deactivate your camera. We encourage you to keep yourself muted when not speaking to avoid background noise and keep the video off to optimize participants' bandwidth.

There are two ways of joining the FGD. **You can log in using the shared Zoom links** (which requires a good internet) or **dial the toll-free number** (which does not require internet connectivity but will require you to input the Meeting ID and Password)**.** If you are disconnected, please join again using one of those methods as soon as possible.

Language

The FGD will be conducted in English and Twi. While the presentation of findings and prompts will be in English, you can respond in English or Twi. Almost all participants of the FGD will be able to comprehend what you say (which is the most important).

Voluntariness

Your participation in the FGD is voluntary, and you can decide to stop participating in the study without explaining yourself. If you choose to withdraw midway during the session, just log out of the Zoom platform using the functions we will demonstrate.

Risks and Benefits

Participation in the FGD could lead to a loss of confidentiality, and some discussions may make you feel uncomfortable. While there is no immediate personal benefit from participating in the study, participating in the FGD could help improve your work environment and patients' hypertension care and outcomes.

Confidentiality

To maintain your confidentiality, the recorded discussion and audio transcripts will be stored on the safe cloud storage approved by the IRB, accessible to the research team members. All

information that could help identify you (including but not limited to your name and the name of the health facility) will be removed from transcripts and quotes to be included in the reports and publications.

Consequence of Withdrawal

We will use the information already collected without any identifier if you decide to withdraw. If you request that we remove your response – please remember that we might not be able to pull it out since we have no way of identifying you from the pool of other responses.

Cost/ Compensation

In appreciation for your time and internet, we will send you 400 GHS on your MTN Mobile Money or Vodafone Cash.

Have questions/concerns?

If you have any concerns about the conduct of this study, your welfare, or your rights as a research participant, you may contact: The Office of the Chairman Committee on Human Research and Publication Ethics.

GROUND RULES

- We recommend not putting video to ensure good internet connectivity.
- Respect other people's ideas and openly voice yours when they are finished talking.
- We encourage active participation.
- Keep focused on the topic or question.
- You are at liberty of using English or Twi.
- For your confidentiality, you can choose not to use your real name and not to mention the name of your health facility

FOCUS GROUP DISCUSSION GUIDE

Warm-up

How do you want us to refer to you during the discussion (state only your first or a fake name to refer to you by)? What is your role at the health facility where you work (you do not have to say which health facility)?

Presentation

1. Mr. Samuel Byiringiro will do a 15-minute presentation of a summary of quantitative findings (availability of trained staff, guidelines, equipment, essential medicines, and leadership support) for hypertension care.
    a. What is your reaction to the presented data?
    b. Do the data reflect the actual situation at your health facilities? Elaborate.
2. In your health facilities, 50% of patients on treatment had uncontrolled hypertension. Based on the services you provide daily, why is that the case?

Service delivery

1. What is a typical process of having a patient diagnosed and initiated into hypertension treatment at your facilities?
2. Half of the surveyed facilities manage patients in hypertension clinics rather than in the general Outpatient Department. We did not find a statistically significant difference in blood pressure control among facilities with hypertension clinics (48% at facilities without and 53% at facilities with hypertension clinics). What are the benefits of managing patients in hypertension clinics vs. in general OPDs?
3. What is the referral system for patients with hypertension (whom do you usually refer to for further management, and to where)?

4. What could be done to make hypertension services more accessible to patients?
5. In the providers’ survey, 65% reported that their facilities had a way of following up on patients with hypertension.
    a. What is your system of patient follow-up?
    b. What are the barriers to having a patient follow-up system?

Healthcare providers

1. Sixty-six percent of providers reported having received training on hypertension, and 11 out of 15 (73%) health facilities reported that at least one of the providers received hypertension training over the last two years.
    a. What level of training is available in your facility?
    b. What provider-level challenges do you face in implementing the hypertension guidelines?
2. How else are providers supported to provide evidence-based hypertension care services?
3. How is the patient load vs. the number of healthcare providers available to manage patients, and how does it impact the quality of hypertension care services?
4. Task-sharing/shifting consists of having non-medical staff trained to manage hypertension patients, including prescribing medications and giving referrals. How is this system practiced in your respective health facilities?
5. Decision support is essential in helping healthcare providers adhere to guidelines. Less than 50% of facilities had hypertension treatment algorithms available in consultation rooms. How are providers supported to access the guidelines while managing patients with hypertension?
6. Peer-to-peer learning among healthcare providers and across health facilities has been shown to improve the quality of health services. Are there such opportunities at your facilities?
7. What could healthcare providers do to improve the quality of hypertension care services?

Financing

1. The National Health Insurance Scheme covered over 99% of sampled patients, and the NHIA covers over 90% of antihypertensive medications. Yet studies reported that the cost of hypertension care was still a barrier to patients' achievement of blood pressure control.

How does the cost of care influence patients' achievement (or non-achievement) of blood pressure control in your facilities?

2. What financing is made available for hypertension (and other CVDs) management activities by the responsible bodies (GHS) and facility management team (for private facilities)?
3. What are other financing barriers to the management of hypertension?

Medicines, tools & equipment

1. Only 60% of facilities had patient education support materials for hypertension. How are patients educated on hypertension/risks of uncontrolled hypertension and recommended lifestyles?
2. Only 9 out of 14 health facilities had all first-line antihypertensive medicines available. What are the barriers to keeping the antihypertensive medication stocks filled?
3. What are the medication-related barriers to managing hypertension?

Health information

1. Discuss the instances of using routine data in improving the quality of hypertension services.
2. Only 60% of providers reported being aware of regular clinical meetings on hypertension care. How are these meetings structured, and why would 40% of healthcare providers not be aware of them? (discussion of data during clinical meetings)

Leadership and governance

1. Most facilities reported having supportive mentorship regarding hypertension care.
    a. Discuss instances in which this mentorship has helped improve the quality of hypertension management at your facilities.
    b. How could the mentorship be improved to further contribute to a better quality of hypertension care?
2. What could health facility leaders do to improve the quality of hypertension care services?
3. What could the Ghana Health Service and the Ministry of Health do to improve the care of patients with hypertension?

   Any additional comments?

Thank you for participating in this focus group discussion!!
