# Supplementary material for: Healthcare system barriers and facilitators to hypertension management in Ghana
Source: Ann Glob Health. 2024 Jul 4;90(1):38. doi: 10.5334/aogh.4246 (PMC11229483; doi:10.5334/aogh.4246)
Supplement: Supplementary File 2. — Table 1. Themes, subthemes, and selected quotes on health system barriers and facilitators of hypertension care in Ghana. [file agh-90-1-4246-s2.pdf]

Supplementary File 2

Table 1. Themes, subthemes, and selected quotes on health system barriers and facilitators of hypertension care in Ghana

Themes	Sub-themes	Barriers of hypertension care – Selected quotes	Facilitators of hypertension care – Selected quotes
1. Financial and geographic inaccessibility of hypertension services	Cost of hypertension care is high	**Cost of care is very high at facilities that do not take health insurance:**<br>"Please in my facility we don't take health insurance. When the patient come, they will pay for consultation, then they will go and do lab investigation as said by the doctor. So, the moment they will realize then their money will be finished." Nurse Assistant<br><br>**Even with insurance though, the user fees (top-ups) make hypertension care more expensive:**<br>"The drugs are quite expensive, and it deters some people even coming for review. We supposed to be covered by national health insurance recently insurance have increased the tax rate, but I don't know whether they have covered fully the cost of management, the cost of the drugs. So, to be frank with you, some pay some amount to top up and somehow deter the patient from coming back." Physician Leader of a Public Health Facility<br><br>"I think for the top up if they can exclude, it will be helpful. Patients mostly come to hospital with the NHIA and mostly are aged. They depend on someone to take care of them. So, if they come and the little money they have has to be used to pay for things, by the time they will be leaving, then the money is finished. They are always complaining." OPD Registered Nurse<br><br>**Participants wish 100% medications coverage by health insurance:**<br>"If the medications can be 100% insured it will really help them because some of them, it is due to the money issues and things get destroyed and that lead to death. All because of the money issues. I'll be happy if something can be done about it." Dietician for Hypertension Clinic	
	There is no financial support system	"There is this particular blind woman, whenever she comes, I have to be the one to pay for her medications but now she is there and so whenever she comes, she says she doesn't have transport money and so you have to give the person some to go." Dietician for the Hypertension Clinic<br><br>"we told her to go for a card and she said she doesn't have money. We called the husband and he said later, so at the end of the day I used my money to go for the card for the patient for the doctor to take care of her." OPD Registered Nurse	

	Limited accessibility of hypertension screening and treatment yet hypertension clinics make a difference	**Screening should be accessible to all people at the health facility**<br>“People are walking through the health facility. They come and visit their patient, I mean they come and take care, the care takers and all those people in our facilities, their BPs must be checked.” Physician Leader of a Public Health Facility<br><br>**Patients who travel long distances struggle to access care**<br>“The thing is, some come from different regions, so sometimes we don’t get to attend to them because of the place they are coming from. That one too is a factor in my facility. Some leave there around 10pm, by that time too, the cardiologist will say I am tired. I must go. So, they wouldn’t even see the cardiologist.” Registered Nurse<br><br>**Hypertension care services are available only a few days in a week**<br>“We have incorporated something we call the hypertension clinical days of which is normally Thursdays of which are chronic patients visits the facility for their monthly anti-hypertensive medications.” Physician Leader of a Private Health Facility	**Wellness clinics help screen people who are not necessarily looking for care**<br>“We happen to pick some of the cases from there [Wellness Clinic]. When we check and you are high more than the standard, we will let you wait for some time, and then we will check it again, if it is still high, then we will refer you to care.” Physician Leader of a Public Health Facility<br><br>**Health facilities in partnership with Healthy Hearts Africa receive support with community outreach**<br>“When the patient comes in at the moment, we have healthy hearts. Which is an organization helping us screen patients and all that, so we go for outreach programs, and we have patient who visits the facility that we screen almost every day, so we are having patients whose BP are either equal to or greater than 140/90, we ask them to come for other checkups on other days.” Outpatient Department (OPD) Registered Nurse<br><br>**Patients managed in hypertension clinic receive education**<br>“As for the clinic, we teach and educate them that there is no cure for hypertension. It is just managed, so the person always has this in their mind, and we always have a talk with them in the morning. So, if the person forgets, he or she will be reminded but OPD isn’t like that.” Hypertension Clinic Registered Nurse<br><br>**Hypertension clinics help minimize the waiting time for patients**<br>“I think that, when it comes to patients coming just to the hypertensive clinic, first of all, I think one of the advantages, is the less waiting time. Physician Leader of a Private Health Facility
	The patient referral system is inconsistent, and patients are reluctant to go to higher (often farther) levels of health systems	**Some health facilities refer if there is no BP control despite lifestyle and medications management and do not have a specialist**<br>“We usually refer where you give them a cocktail of medicines and their BP is still not controlled, very high. you’ve brought in the non-pharmacological aspects in managing… If we don’t have one, then we do refer them to the appropriate specialist.” Physician Leader of a Private Health Facility<br><br>“We start with calcium channel blockers, we’ve given our first line of treatment, so at that point, you’ve given your medications and you are still recording high BP. That means you need to refer to the district level because at the health centers, when we give the calcium channel	**Providers try to learn the patient management at the referral site**<br>“We call the clinician or the receiving facility nurse or the person in charge there to know what exactly they did for the patient. What other challenges they had or something so that we can learn of those things, so basically these are some of the things we also do in the management facility.” Nurse Representative of a Public Health Facility

		blockers, the next thing to do is to refer." Physician Leader of a Private Health Facility<br><br>**District level facilities with a specialist do not refer to higher levels of the health system**<br>"Mostly we manage our patients in the emergency units or our ward. We don't normally refer our patients outside our hospital." OPD Registered Nurse<br><br>**Some patients are referred for medications to be covered by health insurance**<br>Sometimes when you tell them to go there [referral facility] and they will get it [medication] free, they are reluctant. They feel like you are scaring them to go to a facility like Manhyia and they will be like Manhyia is a big facility and I will not go there. OPD Registered Nurse<br><br>**Train more people at sub-district to minimize the need for a referral.**<br>"Clinicians at sub-district level should be trained or should be allowed to manage some levels of hypertension because when you refer them to the district level and sometimes, they have to go and join a long queue, a lot of people, they tend to get their medications from over the counter." Nurse Representative of a Public Health Facility	
	Patient follow-up systems are inconsistent	**Doctors keep changing and patients cannot see the same provider**<br>"And at times a doctor will come there only once a month and will not come again. A different doctor will come, every day we are seeing different doctors." Registered Nurse<br><br>**Follow-up system not fully functional and is an initiative of some healthcare providers instead of facility supported**<br>"I'll give thumbs up to some of my nurses. We look at those who are supposed to come on the Wednesday and then we are supposed to call them. Most often, when you are engulfed in the work for that day, we don't do." Physician Leader of a Private Health Facility<br><br>**Not able to call patients because of lacking credit**<br>"For the calls, at times we are not able to do it due to financial issues. Because if we don't have money to buy credits, it becomes very difficult for us to call them. So that is also one of the barriers." Registered Nurse<br><br>**Patients just purchase meds over the counter and default on follow-up fearing the meds are not available at the hospital**<br>"They [patients] get it [medicine] from over the counter so they don't usually come back for the monthly schedules to take the same medications or to be changed on the medication, so they'll come later with sometimes, complication. they think oh when I come and it's not available, I can get it at the pharmacy so why don't I buy it outside." Nurse Representative of a Public Health Facility	**Patients' follow-up system is available at some facilities**<br><br>"For our facility, when we take your number, we also take your relative's contact or your children's contact or anyone you are staying with or close to you contact. So, if we call you and can't reach you, we call the person and draw the attention of the person." Medical Department Registered Nurse<br><br>**Health facilities in partnership with Healthy Hearts Africa receive support for patient follow-up**<br>"We are supported by the healthy hearts Africa people so every month, they give us an amount of money to buy credits to do follow-ups." Hypertension Clinic Registered Nurse<br><br>"So currently my clinic is working with healthy hearts and then at the end of every month they give us a little money to buy credits to call clients to do follow-ups and all that, but then if there would be any support, we have a lot" OPD Registered Nurse

2. Facilities struggle to maintain the stock of antihypertensive medications and providers perceive government insured medications to be of sub-optimal quality		**Delayed Reimbursement by National Health Insurance Scheme (NHIA) make it challenging to maintain an optimal storage of all essential medications**<br>"You stock the medicines and if reimbursements is also much delayed, then you have to find means of getting money to stock more of the medicines to supply the clients." Physician Leader of a Private Health Facility<br><br>**Yet, NHIA covered medications have so many adverse effects**<br>"Most of the insurance drugs, because of the low quality it has, patients, most of them react to it, its adverse effect." Hypertension Clinic Registered Nurse<br><br>"Another issue is the drugs that the health insurance people provide, most of them are of no quality." OPD Registered Nurse	**Providers reported not having issues with medications**<br>"We hardly get challenges with antihypertensive drugs at our facility. At times, it happens and if it does happen, the pharmacy department will print it out and stamp it for the person to send it to any NHIA facility or pharmacy shop to take it. But it hardly happens." OPD Physician
3. Dire shortage of physicians, and varying roles of clinicians in hypertension management	Dire shortage of physicians	"Sometimes, because of the number of people you must see in a day, there are some people who just come, and you ask if there are any complaints. Some of our colleagues will just take it as if they [patients] came for a refill of their medication without having a critical look at the BP of the patient." Physician<br><br>"So, because of the workload, you see a number of patients; they just want to rush through and be writing their medication." Physician	
	Healthcare provider roles in hypertension management depend on the level of care	**At district hospitals, nurses' roles in hypertension management are limited**<br>"We just check the person's BP, yours is to record what you see on the apparatus then the clinician will take over" Nurse Representative of a Public Health Facility<br><br>**Task-shifting of hypertension roles to non-physician providers is available in rural and hard to reach areas.**<br>"Hard to reach areas in the rural areas, it [task-shifting] works very well, and I think it is being practice also here in Ghana and even in the region where we work. So in the bigger facilities we have a lot of nurses, a lot of physician assistants, a lot of doctors, that is when these things are restricted to only the qualified or let me say to the doctors and the physician assistants." Physician Leader of a Public Health Facility	
	Training opportunities for healthcare providers managing patients with hypertension are scarce, especially for nurses	**The only knowledge sharing opportunities among staff are the unstructured clinical meetings/ which are not necessarily for hypertension**<br>"There are clinical meetings for doctors and in terms of doctor to nurse or doctor to other care of staff, it's not a structured meeting. But there are daily Ward rounds, which involve the nurses and the doctors. That's where the information is shared." Physician Leader of a Public Health Facility<br><br>**There is a need for more training for nurses at the sub-district level in hypertension training**	**Nurses need a training tailored to them specifically – they are eager to have more roles in hypertension management**<br>"If you can organize a special training for nurses specifically for hypertension, I think it will help us. Not for all health care because we will come and will not benefit much from it. Like we will not get access to information." Medical Department Registered Nurse<br><br>"It is just like what nurse "X" has mentioned. You go to sit down and it's all about the doctors and it makes you

		"To also add up, so me personally at the health center or the sub district level, you will have to even if you don't have clinical meeting, you have to train some of your nurses, train almost all of them because you will not always be around, and patients need services all the time, so you'd have to train them." Health facility leader	wonder why you the nurse even went there. So that is the problem we are facing. So just like they are saying if you can organize purposely for nurses only, we will be happy with it." OPD Registered Nurse
4. Patients exhibit both positive and negative self-management practices		**Even when they take medications, they keep unhealthy lifestyles** "So, they only take the drug, and they still eat late, fatty foods, they don't exercise, they have this sedentary work." Registered Nurse in the General Department **And patients opt for traditional medicines** "Where they have some myths that herbal preparation can cure it so they come, you give them the drugs, they go and dash it somewhere and they are taking the herbal medication" Physician Leader of a Private Health Facility<br><br>"Some think having hypertension is the doing of witchcraft or superstition, so they will take to church for prayers and not come to the hospital." Hypertension Clinic Registered Nurse<br><br>**Some patients expect hypertension to be fully cured by prayer** "We try to advise them that since the pastor is doing the spiritual aspect, do both so that at all times to control your blood pressure." Dietician at a Hypertension Clinic	**Patients have BP machines at home** "Most hypertensive patients have BP machines at their home so when the doctors go there, they take advantage of that and examine you, your lifestyle and what contributes to making your BP raise faster and what causes you to have reactions all the time." Health care providers
